# Forecasting the effectiveness of the DeWorm3 trial in interrupting the transmission of soil-transmitted helminths in three study sites in Benin, India and Malawi

**DOI:** 10.1186/s13071-020-04572-7

**Published:** 2021-01-20

**Authors:** James E. Truscott, Robert J. Hardwick, Marleen Werkman, Puthupalayam Kaliappan Saravanakumar, Malathi Manuel, Sitara S. R. Ajjampur, Kristjana H. Ásbjörnsdóttir, Kalua Khumbo, Stefan Witek-McManus, James Simwanza, Gilles Cottrell, Parfait Houngbégnon, Moudachirou Ibikounlé, Judd L. Walson, Roy M. Anderson

**Affiliations:** 1 London Centre for Neglected Tropical Disease Research, Department of Infectious Disease Epidemiology, Imperial College London, St Mary’s Campus, London, W2 1PG UK; 2 MRC Centre for Global Infectious Disease Analysis, Imperial College London, St Mary’s Campus, London, W2 1PG UK; 3 The DeWorm3 Project, The Natural History Museum, London, SW7 5BD UK; 4 Julius Center for Health Sciences and Primary Care, University Medical Center Utrecht, Utrecht University, Utrecht, The Netherlands; 5 The Wellcome Trust Research Laboratory, Division of Gastrointestinal Sciences, Christian Medical College, Vellore, India; 6 Departments of Global Health, Medicine (Infectious Disease), Pediatrics and Epidemiology, University of Washington, Seattle, WA USA; 7 Blantyre Institute for Community Outreach, University of Malawi, College of Medicine, Blantyre, Malawi; 8 Faculty of Infectious and Tropical Diseases, London School of Hygiene & Tropical Medicine, London, UK; 9 Institut de Recherche pour le Développement, MERIT, Université de Paris, Paris, France; 10 Institut de Recherche Clinique du Benin, Université d’Abomey-Calavi, Cotonou, Benin; 11 Département de Zoologie, Faculté des Sciences et Techniques, Université d’Abomey-Calavi, Cotonou, Benin

**Keywords:** Soil-transmitted helminths, Transmission interruption, Individual-based simulator, Heterogeneity

## Abstract

**Background:**

The DeWorm3 project is an ongoing cluster-randomised trial assessing the feasibility of interrupting the transmission of soil-transmitted helminths (STH) through mass drug administration (MDA) using study sites in India, Malawi and Benin. In this article, we describe an approach which uses a combination of statistical and mathematical methods to forecast the outcome of the trial with respect to its stated goal of reducing the prevalence of infection to below 2%.

**Methods:**

Our approach is first to define the local patterns of transmission within each study site, which is achieved by statistical inference of key epidemiological parameters using the baseline epidemiological measures of age-related prevalence and intensity of STH infection which have been collected by the DeWorm3 trials team. We use these inferred parameters to calibrate an individual-based stochastic simulation of the trial at the cluster and study site level, which is subsequently run to forecast the future prevalence of STH infections. The simulator takes into account both the uncertainties in parameter estimation and the variability inherent in epidemiological and demographic processes in the simulator. We interpret the forecast results from our simulation with reference to the stated goal of the DeWorm3 trial, to achieve a target of $\le 2\%$ prevalence at a point 24 months post-cessation of MDA.

**Results:**

Simulated output predicts that the two arms will be distinguishable from each other in all three country sites at the study end point. In India and Malawi, measured prevalence in the intervention arm is below the threshold with a high probability (90% and 95%, respectively), but in Benin the heterogeneity between clusters prevents the arm prevalence from being reduced below the threshold value. At the level of individual study arms within each site, heterogeneity among clusters leads to a very low probability of achieving complete elimination in an intervention arm, yielding a post-study scenario with widespread elimination but a few ‘hot spot’ areas of persisting STH transmission.

**Conclusions:**

Our results suggest that geographical heterogeneities in transmission intensity and worm aggregation have a large impact on the effect of MDA. It is important to accurately assess cluster-level, or even smaller scale, heterogeneities in factors which influence transmission and aggregation for a clearer perspective on projecting the outcomes of MDA control of STH and other neglected tropical diseases.

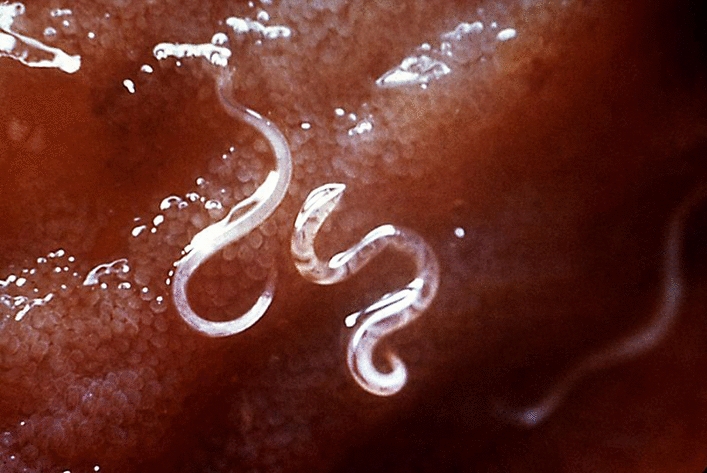

## Background

Soil-transmitted helminths (STHs) are a group of parasitic worms that infect humans, causing a wide spectrum of disease, notably anaemia, growth retardation and delayed cognitive development in children. The three main STHs are *Ascaris lumbricoides*, *Trichuris trichiura* and hookworm (*Necator americanus* and *Ancylostoma duodenale*). Approximately 1.5 billion people are currently thought to be infected with STHs worldwide. The World Health Organisation is targeting morbidity control, defined as reaching $<2\%$ prevalence of medium-to-high intensity infections (as detected using coprological approaches such as Kato-Katz) in preschool-age children and school-age children (SAC) as a goal for 2030 [1]. Treatment guidelines for achieving this goal are under revision at present with respect to the creation of a new 2020–2030 roadmap for the WHO NTD control programme. The overall goal is likely to be similar to the 2010–2020 roadmap with emphasis on morbidity control in both children and women in the pregnancy age classes in areas hookworm is endemic.

**Fig. 1:**
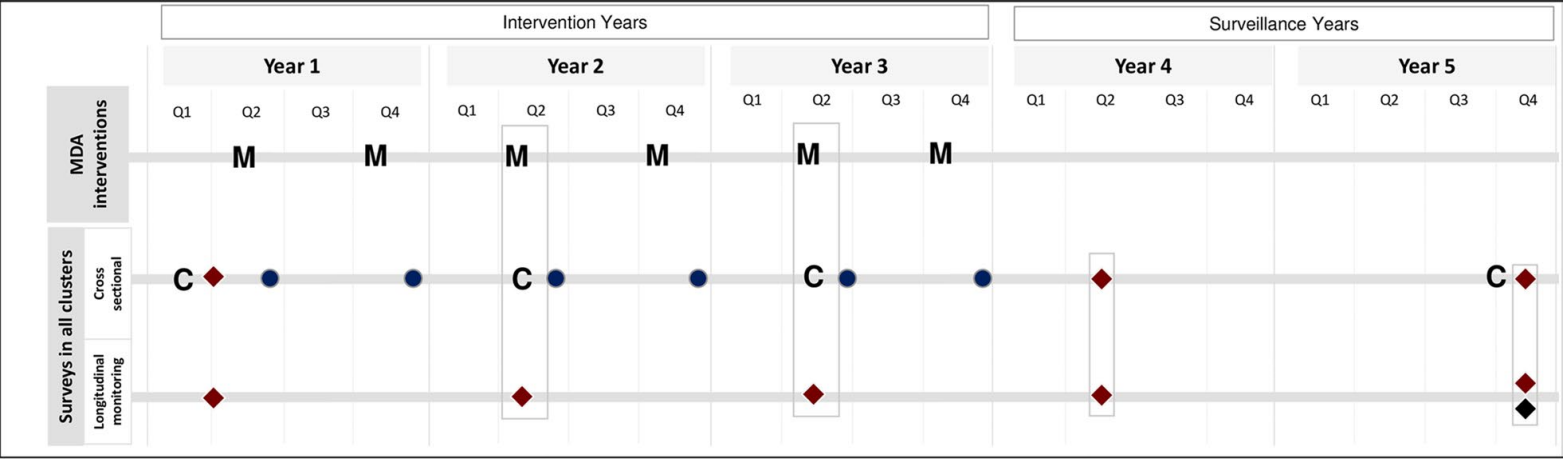
Timeline of the DeWorm3 trial intervention arm. ‘M’s indicate the six rounds of MDA with red diamonds representing stool collection events in the trial population as a whole and the longitudinal cohort. Symbols: ‘C’: census; blue circles: post-treatment coverage surveys, blue diamond: blood spot collection (Figure adapted from [3])

The DeWorm3 trial is designed to test the feasibility of moving beyond morbidity control with the focus on breaking the transmission of STH diseases using community-wide mass drug administration, targeting all ages (MDA). It is constructed to work at two spatial scales. At a large scale, DeWorm3 tests whether community-wide MDA can succeed at the level of the study arm. Additionally, it is powered to examine how the outcome at the arm scale is reproduced at the level of individual clusters within an arm. Spatial heterogeneity on a range of scales is a key feature of STH infection [2]. It also seeks to ‘leverage’ the impact of past and present MDA programmes to control lymphatic filariasis using albendazole which is also a drug of choice to treat STH infection. The trial comprises study sites in India, Benin and Malawi and is described in full elsewhere [3, 4]. In each location, a cluster-randomised trial is conducted with 40 clusters assigned to one of two arms. In the intervention arm, clusters are subject to twice-yearly MDA over 3 years (see Fig. 1). The control arm receives standard of care according to national guidelines for STH control, which vary somewhat between country sites [3]. In both arms, all chemotherapy is suspended between the final round of MDA and the end line survey. For the purposes of the trial, breaking transmission for an STH species is defined as reaching a prevalence of $\le$ 2%, as detected by PCR, at a point 24 months after the final round of MDA. The threshold of 2% has previously been shown, through modelling work, to have a high positive predictive value (PPV) (approximately 80%) as a predictor of ultimate parasite transmission elimination for STH [5]. The trial also aims to demonstrate that the intervention is significantly better at reducing STH prevalence than the national standard of care for STH infection in each country.

The analyses presented in this article use individual infection intensity data from the trial baseline survey and compliance data from the first two post-MDA coverage surveys to parameterize a stochastic individual-based simulation model [4]. Individual-level baseline data (anonymised with respect to individual identity and stratified by country, arm and cluster) allow us to capture the spatial heterogeneity in the prevalence and intensity of infection within the study sites. The dominant infection is hookworm across the three country sites, with *Ascaris* being significant in only a few clusters in the Benin site. As a result, we confine the simulation to hookworm only. The simulation is used to run the trial forward *in silico* for all the clusters in the two study arms in each of the sites. MDA rounds and cross-sectional surveys are simulated, and the simulation is then run on to 20 years beyond the study endline to examine if transmission breaking and parasite elimination are predicted to be achieved.

The purpose of the simulation is to forecast the impact of the trial intervention at the key survey time points described in the study protocol and also to look at the longer term effect of the study on disease burden in the population. The major reason for doing this is to help identify the most appropriate analytical methods and to assist in the interpretation of the trials results. Key questions we address are:

- To what extent is elimination achieved in the long term, beyond the end point of the study, at the cluster, study arm and country site level? The individual-based model allows us to address indicators of true elimination (the total absence of parasites in the host population) of disease in the population. In this work, the indicator used is the absence of female worms in the sampled population.
- How does the cluster-level baseline heterogeneity in prevalence and intensity of infection affect the possibility of elimination?
- Is the 2% prevalence threshold, 24 months post-treatment, an effective test for the long-term breaking of transmission?
- Is the 2% threshold equally effective at the arm level and at the level of individual clusters?
- Can the two arms of the study be clearly distinguished in terms of the reduction in the prevalence of infection achieved, given the observed patterns of infection prevalence and intensity of infection recorded at baseline?

## Methods

Model forecasts are generated by an individual-based trial simulator. The structure of the stochastic model is based on published work defining the deterministic framework of STH transmission models and associated estimates of key population dynamic parameters such as parasite life expectancy in the human hosts [6, 7]. Since hookworm is the dominant STH infection in all three study sites, we focus the following analyses on this parasite.

The simulator is initialised using the posterior distribution of parameters fitted to the baseline data and generates ensembles of individual-level time series of parasite burden, stratified by age, cluster, study arm and site. We allow the simulations to proceed beyond the trial endline to forecast what will happen in the longer term, in particular, whether elimination of the parasite from the population will be achieved. The ensemble of virtual trials also includes the variability arising from parameter value uncertainty and from epidemiological and demographic processes such as worm acquisition, worm death, host birth and death as well as treatment and finite population variance. Ensembles are analysed to examine patterns of prevalence and intensity and their variability.

The trial simulations are not identical in structure across the three sites. Standard of care for STH varies between countries in terms of treatment frequency, timing and the age group targeted, leading to differences in the control arms. As a result, the ‘meshing’ of the DeWorm3 treatment period with a country’s standard of care activities also varies, leading to variation across the intervention arms. Details of the timelines in each country are described extensively in Additional file 1.

We examine the the following aspects of the projected disease state of the study area.

- Time series information; how does the measured prevalence and intensity of infection vary through the course of the study? What is the character of the geographical heterogeneity? How does it behave after the study has ended?
- Elimination of disease; we look at the probability of elimination of hookworm from the whole population and also the pattern of elimination within individual clusters. How does this depend on key epidemiological parameters like $R_0$ and *k*? How good is an end point threshold prevalence as an indicator of eventual elimination?

### Infection transmission simulator

The model incorporates the basic epidemiological processes of the acquisition of worms through contact with an infectious reservoir and the death of worms within individuals. These processes are represented as stochastic and individual-based at the level of individuals and the worms within them and are described in Table 1. The force of infection from the infectious reservoir, $\lambda _i(a,t)$, is made different for each individual by a multiplicative factor $\eta _i$, such that the force of infection for the acquisition of female worms in individual *i* at age $a_i$ and time *t* is given by

1 $$\begin{aligned} F_i(a,t) = \eta _i\beta (a_i)L(t) \end{aligned}$$

The parameter $\eta _i$ is drawn from a gamma distribution with shape parameter 1/*k* and mean 1, $\eta _i \sim \Gamma (1/k, 1)$. In this way, the equilibrium distribution of worms in the host population will have a negative binomial distribution as observed. Infection rate in individuals is controlled by an environmental reservoir of infectious material.

2 $$\begin{aligned} \frac{\text{d}L}{\text{d}t} = \psi - \mu _2L \end{aligned}$$

where

3 $$\begin{aligned} \psi = \frac{\phi }{N}\sum ^N_i w^F_i\exp \{-\gamma w^F_i\} \end{aligned}$$

In the above, $\phi$ controls the transmission intensity of the disease and is a function of the other model parameters and $R_0$. The parameter *N* is the number of individuals in the cluster and is included to make the model frequency- rather than density-dependent, that is, transmission intensity is not a function of cluster population size.

**Table 1:** Event table stochastic processes related to individual *i* with age $a_i$, infected with $w^T_i$ worms in total with $w^F_i$ females

Mechanism	Rate	Event
Worm acquisition	$2F_i(a,t)$	$w^T_i \rightarrow w^T_i + 1$,
		$$w^F_i \rightarrow w^F_i + \text{ Bern }(0.5)$$
Worm death	$\sigma$	$w^T_i \rightarrow w^T_i + 1$,
		$$w^F_i \rightarrow w^F_i + \text{ Bern }(w^F_i/w^T_i)$$
Host birth/death	$\mu (a)$	$a_i \rightarrow 0;\, w^T_i, w^F_i \rightarrow 0$
Host compliance at MDA round *j*	–	Treat with $P = c^j(a_i)$
Treatment of compliant individual	–	$a \sim \text{ Bin }(w^T_i-w^F_i, 1-e_f)$
		$$b \sim \text{ Bin }(w^F_i, 1-e_f)$$
		$$w^T_i = a+b,\, w^F_i = b$$

Each individual is characterised by a value $\eta _i$ that describes their individual strength of contact with the infectious reservoir. $F_i(a,t)$ is the force of infection for the acquisition of female worms in individual *i* (see Eq.1)

Treatment during MDA is modelled as a random process at an individual level. At each round of MDA, whether an individual is treated is the result of an independent Bernoulli trial with a probability of success given by the treatment coverage in that country at baseline. Probability of treatment at MDA is therefore dependent on age (SAC or adult) and country site, but not on MDA round or past behaviour. Coverage levels for both the intervention and control arms are estimated from associated DeWorm3 survey data as described in Additional file 2. This assumption is the simplest and also the most optimistic in terms of MDA impact. In reality, there may be significant correlations between individuals’ adherence to treatment from round to round or heterogeneity in adherence within an age group and country site. These mechanisms tend to increase the probability that individuals avoid being effectively treated. Data to inform more complex models of these types are being collected in the course of the current trial.

The simulator generates results in terms of the internal worm state of each individual in a cluster. As such, the ‘true’ prevalence of the parasite in the host population can be calculated (whether this is the prevalence of individuals having at least one worm, at least one female worm or at least one fertilised female worm). To generate results equivalent to those described in the protocol, we construct a samples of 1000 individuals from each cluster at the end point [3]. A diagnostic model is used to determine if an individual in the sample is parasite-positive. We consider both the standard Kato-Katz method and the DNA-based qPCR method [8, 9] (see Additional file 3 for diagnostic model details). Kato-Katz is the diagnostic used for the baseline data to which the model is fitted and PCR is the diagnostic chosen for the endline prevalence survey. A key advantage of the simulator is the ability to estimate when parasite elimination has occurred in a sub-population. In the following, we assume long-term elimination to have occurred in a cluster if, at a time point 10 years after the endline, there are no individuals in that cluster that have any female worms.

### Parameterization

The parameters for the simulator can be grouped into two sets: global and local parameters. Global parameters describe fundamental features of STH epidemiology and were assumed be shared by all individuals across all clusters and all country sites. Values were taken from sources in the literature or past fitting work. Local parameters relate to processes which depend on local social structure demography. In particular, we focus on the degree of worm aggregation among the host population, the relative age-dependence of the force of infection and the overall transmission intensity, as represented by $R_0$. Local parameters were estimated at the cluster level from DeWorm3 baseline data. The details of the fitting process and parameter value sources are found in Additional file 3.

We constructed a deterministic version of the disease transmission model within the trial simulator and used it to construct a likelihood for the baseline egg count data for each study cluster in each country, assuming an endemic state in each cluster. The technical details and results are described in Appendix Additional file 3. A single cluster with no measured disease at baseline was omitted. Prior distributions on parameters were used to ensure stable disease states were generated and that very high worm individual burdens (more than 80 worms per individual) were very unlikely. Standard Monte Carlo Markov chain techniques were used to sample from the posterior distribution, capturing the uncertainty in parameter estimates as well as any correlations that might exist between them. Samples from the posterior distributions were used as a source of parameters for the stochastic simulation. The clusters within each study arm were parameterized with samples from their respective posteriors for each of 200 iterations.

## Results

### Data summary

Some of the key features of the data are summarised in Table 2. The Indian study population stands out as having the highest baseline prevalence at 16.6%. However, the range of prevalence across individual clusters is wide, spanning 60% down to a fraction of a percent. This is a clear indication there is considerable geographical heterogeneity in this study site.

**Table 2:** Summary statistics of baseline data by country across both study arms

Country	India	Malawi	Benin
Overall prevalence	16.6%	7.5%	3.5%
Cluster prevalence range	0.7−60%	1–19%	0.6–21%
Overall mean egg count	4.4	1.5	0.8
Range of mean cluster egg counts	0.04–27	0.02–7.1	0.006–7.6

Mean cluster ranges quoted are absolute ranges across all clusters and mean egg counts are across all tested individuals, not only egg-positive ones. Only hookworm data are shown

### Parameter distributions

**Fig. 2:**
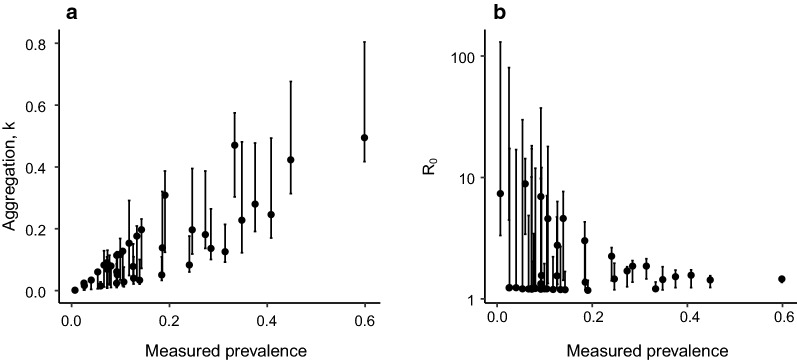
Posterior distributions for negative binomial aggregation parameter, *k*, and reproduction number, $R_0$, for individual clusters, derived from baseline data at the India site. Bars represent the 95% credible interval for each inferred value. (Equivalent distributions for other country sites found in Additional file 3)

Figure 2 shows the maximum likelihood estimates (MLEs) and 95% credible intervals for the aggregation parameter *k* and the reproduction number $R_0$ fitted independently for each cluster at the India site (other country site distributions are shown in Additional file 3. Parameter distributions are qualitatively similar to those from the other baseline survey fits, although parameter magnitudes differ. A key feature of the distributions is the strong linear correlation between the measured prevalence and aggregation parameter, *k*. As baseline prevalence decreases, the degree of aggregation increases (as indicated by a decreasing value for *k*). This effect was also observed when fitting to cluster-level data from the baseline of the recent Tumikia study in Kenya and with a very similar slope [2, 10]. Greater aggregation concentrates the worms in fewer individuals, leading to a smaller prevalence for a given overall worm burden. The concentration of worms in fewer individuals also requires a larger $R_0$ to maintain disease transmission in the population as a whole. Consequently, $R_0$ values tend to increase as *k* decreases. The large uncertainties associated with the $R_0$ estimates in Fig. 2 reflect the large variability in individual egg count from a given worm burden. At high aggregation, few individuals give a non-zero egg count and consequently the effect of the egg count variability is magnified.

### Model results

#### Study arms: endline prevalence statistics

Prevalence at the endline is measured from a sample of 1000 individuals per cluster and an average prevalence for an arm can be calculated by averaging the prevalences across all the clusters in the arm. Within the simulation, we construct multiple (200) realisations of each study arm by sampling from the realisations of clusters in the arm and then sampling 1000 individuals within each cluster, using a PCR diagnostic model, to get the sampled prevalence in each arm. There is currently no widely accepted model for the relationship between worm burden and a positive PCR test result. The protocol of the test involves the bead-beating of stool samples to release genetic material from eggs, which are then detected by the PCR method [8, 11]. Hence, the PCR diagnostic method can be viewed as an egg detection technique, but with greater sensitivity than Kato-Katz. Here, we model the PCR diagnostic technique as a test for the presence of fertilized female worms within the host with 100% sensitivity. This probably represents the maximum sensitivity that would be possible using PCR diagnostics in a real-world situation.

**Fig. 3:**
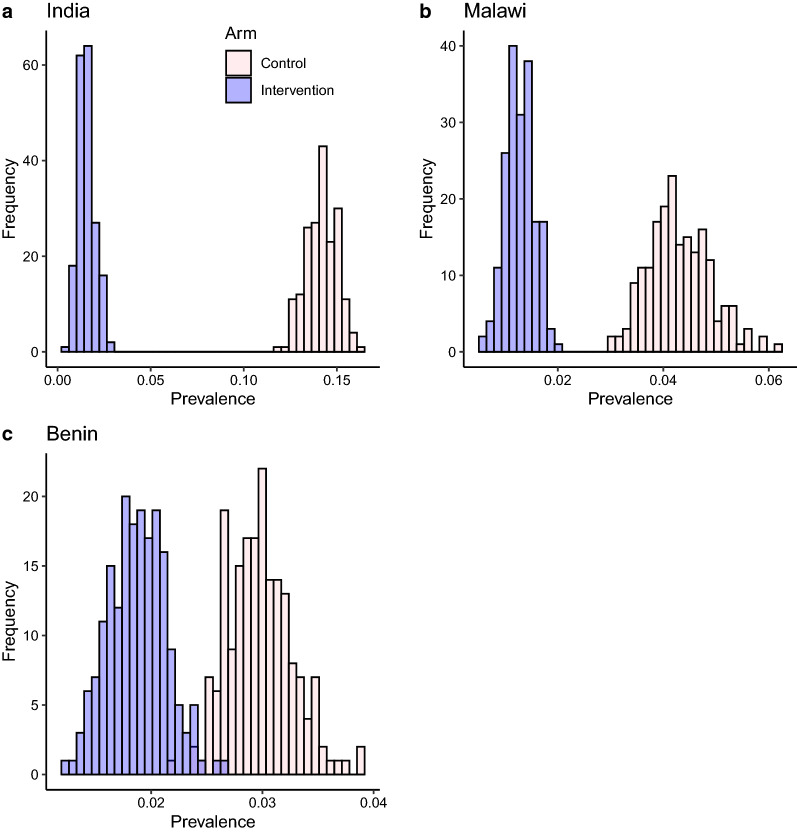
Histograms of sampled prevalence in study arms in each study site at endline. The diagnostic model is PCR

Figure 3 shows that the distinction between the two arms is very clear in the case of India and Malawi, but less so in the case of Benin. The distribution of sampled prevalence is significantly below the 2% elimination threshold discussed in the original protocol for India and Malawi (90% and $>95\%$, respectively). In the case of Benin, however, the 2% threshold lies within the sampled prevalence distribution of the intervention arm (while clearly below that of the control arm). As we will explore in the "Discussion" section, the variance observed in the sampled arm prevalence is largely a reflection of the underlying heterogeneity among clusters in an arm. Cluster-level heterogeneity also has a strong effect on the ability of MDA to bring about true elimination of the parasite in an arm, as defined in the section on the transmission simulator.

#### Cluster level: prevalence drop across the trial

**Fig. 4:**
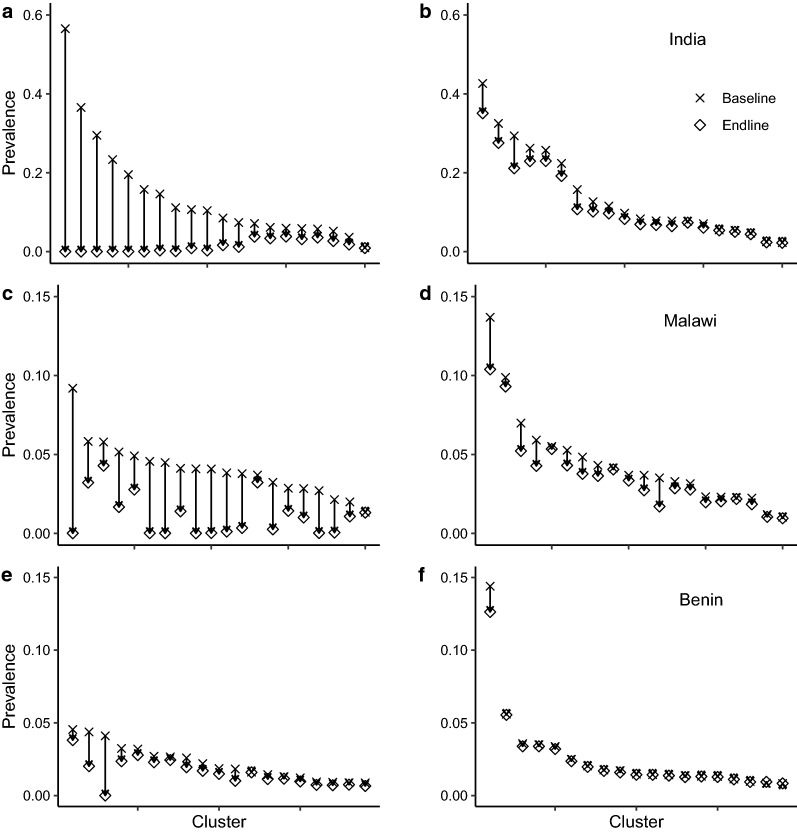
Drop in mean prevalence between the baseline and end point of the trial by cluster. Each vertical line represents the change in prevalence between the baseline and endline of a cluster. Clusters are ordered by baseline prevalence. Panels are stratified by country (rows) and study arm (columns). Diagnostic is Kato-Katz at baseline and endline for consistency

Figure 4 illustrates the effectiveness of the intervention and control arms at reducing prevalence stratified by country and study arm. Each vertical arrow shows the drop in prevalence in a particular cluster in the study arm between the two time points (ordered by baseline prevalence). Prevalence values shown are the means across realisations. The figure clearly shows the heterogeneity in baseline prevalence, with the majority of clusters grouped around lower prevalences and a minority with higher prevalences, giving a positively skewed distribution shape common to all sites. Also clear is the difference in the impact on prevalence of the two arms. Generally, the control arm with standard of care only causes a limited reduction in the prevalence across the study, although there is some clear variation; standard of care in Malawi appears to be relatively effective at prevalence reduction.

In comparing the three country sites, there is a clear relationship between the impact of the intervention and the mean prevalence at a site. In India, the sites with the highest baseline prevalence experience the greatest drop in prevalence across the study, while Benin, with prevalences all < 5%, experiences the smallest impact. Some of this effect may be accounted for by the higher coverage in India than in Benin or Malawi, but this difference is relatively small. More important is the difference in epidemiological parameters between sites. As explained in the "Methods" section, baseline parameter fitting showed that lower prevalences were associated with higher degrees of aggregation. With worm populations more highly aggregated, it is more difficult for all worms in all individuals to be killed, given imperfect drug efficacy and limited coverage.

**Fig. 5:**
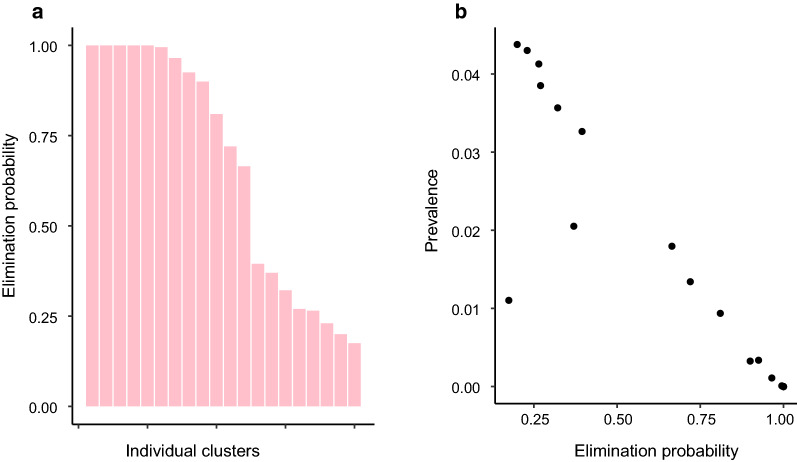
Behaviour of clusters in the intervention arm in India. **a** Probability of elimination at 10 years post endline for each cluster in the study arm. **b** Elimination probability against mean prevalence at endline for each cluster. PCR Diagnostic at endline

**Fig. 6:**
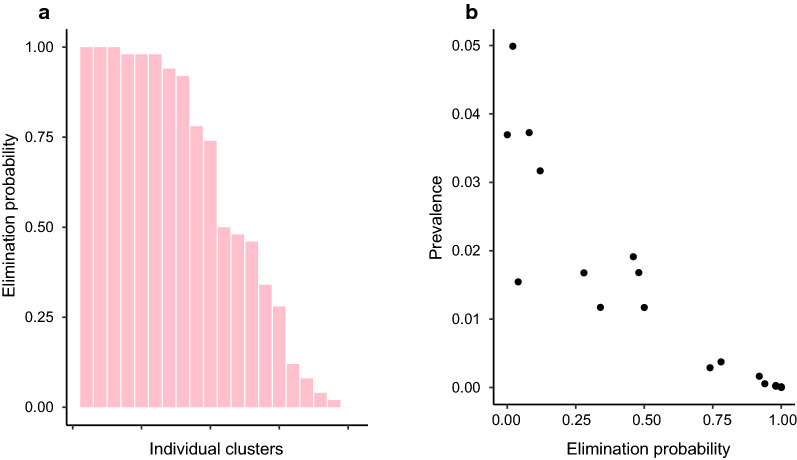
Behaviour of clusters in the intervention arm in Malawi. **a** Probability of elimination at 10 years post endline for each cluster in the study arm. **b** Elimination probability against mean prevalence at endline for each cluster. PCR diagnostic at endline

**Fig. 7:**
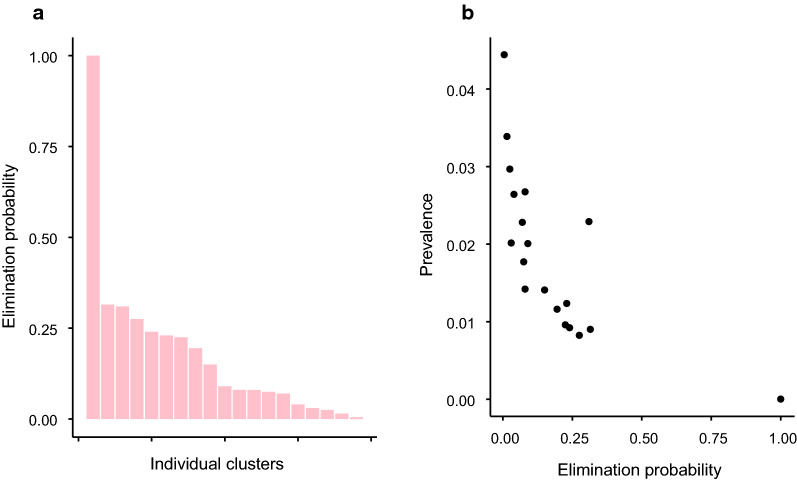
Behaviour of clusters in the intervention arm in Benin. **a** Probability of elimination at 10 years post endline for each cluster in the study arm. **b** Elimination probability against mean prevalence at endline for each cluster. PCR diagnostic at endline

Figure 4 indicates that, particularly in India and Malawi, the MDA intervention reduces the predicted prevalence for many clusters too close to zero at the endline. Figures 5, 6 and 7 show how the expected endline prevalence is related to true elimination of infection 10 years after endline, as defined in the "Methods" section. While elimination in the whole intervention arm is very unlikely, the probability of elimination in individual clusters is highly variable. In India (Fig. 5) and Malawi (Fig. 6), approximately a third have elimination probabilities > 90%. Roughly a quarter of clusters have an elimination probability < 0.25. Elimination probability is strongly correlated with endline prevalence, with the probability of infection *persisting* roughly proportional to the endline prevalence (Figs. 5b, 6, 7b). The cluster elimination profile of Benin is different from those in the other two sites. The high levels of parasite aggregation mean that all but one of the clusters have elimination probabilities < 30%. The relationship between elimination probability and endline prevalence is also qualitatively different from the other two sites.

**Fig. 8:**
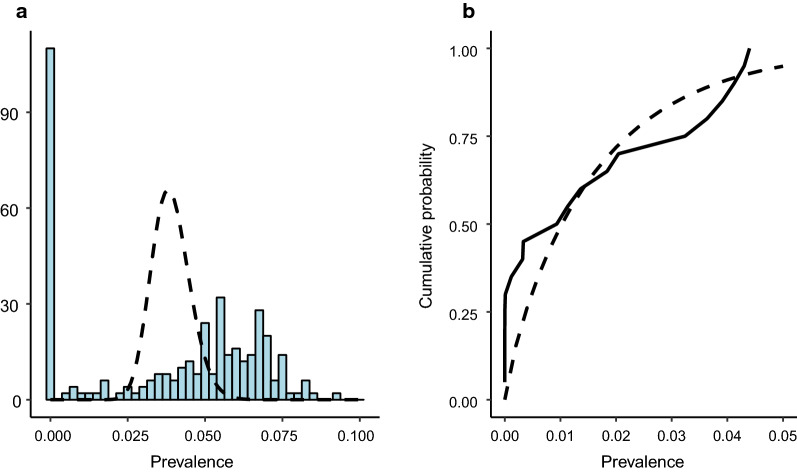
**a** Prevalence variability in a single cluster in the intervention arm in India across multiple realisations. Dotted line represents the estimated prevalence uncertainty for each cluster, based on a binomial assumption with the same mean. **b** Cumulative distribution of mean cluster prevalence across multiple realisations (solid line) and best fit cumulative beta distribution. PCR diagnostic at endline

The standard study design has no knowledge of the ‘internal’ dynamics of clusters and is forced to assume that the prevalence of a cluster is the product of a binomial process with a constant probability of infection for all individuals in a given age category. Uncertainty in prevalence estimates are therefore derived from the sampling error from a binomial process. The simulator includes the variability of epidemiological processes and the uncertainty in the governing parameter values as well. As a result, the variability in prevalence across realisations of the same cluster is much greater than would be expected from a binomial sampling process. Figure 8a shows the variation in the sampled prevalence value across realisations of one of the clusters from the intervention arm in India. The dashed lines represent the sampling uncertainty expected from a classical binomial sampling process with mean given by the mean prevalence of the simulations. It is clear that the predicted distribution of prevalence from the simulation has a much larger variance and also has a different shape. In particular, the classical assumption would do particularly poorly at predicting whether elimination had been achieved in a cluster. In reality, the placement of the mean for the binomial assumption would be based in a single prevalence estimate at the end point, a value drawn from the simulated prevalence distribution shown in blue. As such, decisions based on such an assumption could be highly unreliable.

Sample size calculations for cluster-based trials are often based on the assumption that the distribution of prevalence across clusters is beta-distributed. Figure 8B shows the cumulative distribution for mean prevalence in the clusters of the intervention arm in India at the trial end point, constructed from simulator output, compared to the best-fit beta distribution to the same data. Although the sample is sparse, the beta distribution appears to give a reasonably good fit to the data, suggesting that a beta distribution is a good description of the overall distribution. However, note that the fit is poor for the lowest prevalence clusters. This implies that the beta distribution gives a poor prediction of the probability of the lowest prevalence clusters and these are the clusters in which elimination is most likely to have occurred or to occur in the future. A key parameter describing the variance among clusters is the intra-cluster correlation coefficient (ICC), necessary, along with the mean prevalence in the arms, for sample size calculations. Additional file 4 examines how the value of ICC varies across the duration of the trial under the influence of MDA.

## Discussion

Simulation of the DeWorm3 trial employing an individual-based stochastic model of transmission and MDA impact suggests that the two primary outcomes of the DeWorm3 trial will be largely achieved, but with a few important provisos [3]. At all three sites, the sampled arm prevalence distributions for the intervention and control arms were distinguishable, although in the case of Benin, the resolution was marginal. In Malawi and India, the probability of the sampled prevalence in the intervention arm being below the threshold of 2% was 90% and $>95\%$, respectively. In the case of Benin, the 2% threshold lay well within the range of possible sampled prevalence values in the intervention arm (see Fig. 3). The source of the variability in prevalence is almost entirely the variability in prevalence across clusters and is therefore a feature of the spatial heterogeneity in epidemiological and demographic processes. As such, it cannot be reduced by increasing the sample size in each cluster. On the contrary, in this regard we found very little sensitivity to the sample size and that the distribution of sampled prevalence was unaffected by reducing the sample size to 500 or even lower.

At the level of clusters, the influence of spatial heterogeneity is more clear. The baseline distribution of prevalence for all sites in the data is broad, and the mean prevalence in each site differs widely (see Table 2).

Heterogeneity in baseline clusters data translates to a heterogeneity in the fitted key epidemiological parameters such as the reproductive number $R_0$ and the degree of parasite aggregation as measured inversely by the negative binomial parameter *k* (see Fig. 2). The key feature in the fitted parameter distributions is the strong linear correlation between baseline prevalence and and aggregation parameter *k*, with lower baseline prevalence associated with higher aggregation. Higher aggregation is in turn associated with higher transmission intensity (as represented by $R_0$), since the smaller number of infected individuals needs to compensate to support the transmission process in the host population. The combination of these two effects means that when prevalence is low, transmission is supported by fewer infected individuals with higher rates of transmission. These individuals may be hard to reach with broad MDA with imperfect coverage and hence lower prevalence populations become less responsive to treatment. This is the mechanism behind the apparently counterintuitive result that clusters with lower baseline prevalence are less affected by treatment than those with higher prevalence. This cannot be assessed at present from the trial data but such information will be available when the trials ends.

The relationship between endline prevalence and eventual elimination of parasites in the population indicates that, even though the elimination threshold may have been met at the arm level, the probability of elimination across an entire arm is very low. However, when individual clusters are considered, there is a broad range of elimination probabilities with approximately half of clusters having a > 50% chance of elimination in India and Malawi. For elimination within an arm, all constituent clusters must achieve elimination, and this becomes increasing unlikely as the number of epidemiological independent clusters increases. It is not clear how many independent transmission units (ITUs) comprise the study arms within the trial or, on a larger scale, what is the ‘granularity’ of independent transmission units in a district or region of a country. On the one hand, if the mechanism of transmission is much more household-based than our current model allows, we would expect many more ITUs and a consequent increase in the difficulty of achieving large scale elimination [12]. On the other hand, our model does not include the effects of movement, which will clearly increase the spatial correlation of the infection process, leading to larger ITUs and making elimination easier [13, 14]. As such, our current model at the cluster level probably represents an intermediate description of the impact of MDA in achieving elimination. The results of our current forecasting certainly suggest that the impact of MDA is not only to bring about a large overall reduction in the prevalence of STH, but also to generate a much more heterogeneous distribution of infection spatially. This is entirely consistent with the existence of infection ‘hot spots’ that have been observed for STH and other parasitic diseases in the aftermath of protracted regimes of MDA [15, 16]. In consequence, we believe that simulators of the current type can play a role in understanding how spatial heterogeneity of infection develops under MDA and hence in what kind of monitoring and interventions will be most effective and efficient in eliminating the last pockets of parasitic infection.

Classical statistical methods for sample size calculations are based on key assumptions about the distribution of prevalence both within and between clusters. However, Fig. 8 suggests that the combination of parameter uncertainty, spatial heterogeneity and non-linear parasite transmission dynamics leads to different, non-standard distributions. The distribution of between-cluster prevalence is reasonably described by a beta distribution, but that distribution does not reflect the proportion of clusters that have achieved very low or zero prevalence. Within-cluster variance is also much higher than binomially distributed cases in a sample would suggest, with the distribution often having a bimodal form. This suggests that the standard calculations about where the quantiles of these distributions fall, as used in the calculations of sample size and power, may not be reliable. Additionally, given that the purpose of many studies is to investigate the possibility of achieving very low prevalence or elimination at a cluster or arm level, predictions of these types of outcomes using standard statistical approaches may be particularly prone to inaccuracy.

There are a number of the assumptions that underlie these model-based predictions that need to be considered when evaluating the accuracy of the results. It is assumed that STH infection is in a stable endemic state at the baseline survey, in all cluster and all country sites. However, it is possible that, in some clusters, levels of STH might be recovering from recent national intervention programmes (either for LF- or school-based STH deworming programmes), resulting in an underestimate of the pristine transmission intensity within a defined site [17]. It is also the case that close to the stability breakpoint for the parasite, dynamics can be very slow, leading to apparently stable populations that are far from their equilibria [6, 14]. While it is not clear that these represent a particular bias, they will likely increase the variability in the simulator results. A further unknown is whether the worm aggregation characteristics of a host population are constant over time. It is clear from model fits to the individual clusters that there is a strong linear relationship between prevalence and fitted worm aggregation as measured inversely by the negative binomial *k* value (Fig. 2). A very similar relationship exists for the fit to baseline data from the Tumikia trial [2]. It is not yet clear whether worm aggregation will change in a given population as prevalence is reduced by treatment, perhaps because of persistent non-compliance to treatment by a small segment of the population. If aggregation of worms is increased by MDA, elimination would be even more difficult than found using the current transmission model structure. Our assumptions about MDA coverage have been based on early coverage surveys. However, evidence from more detailed treatment surveys suggests that estimates from coverage surveys may be overestimates (the disparity resulting from proxy reporting, recall bias, and the lack of direct observation of treatment). As a result, our assessment of the impact of MDA may be optimistic. We have also assumed a random pattern of compliance for individuals, whereas information from early rounds suggests that there may be heterogeneity in individual compliance, with some being consistently less likely to receive treatment than others. Such individuals can effectively 'carry’ the parasite through rounds of MDA, leading to less impact for MDA than coverage levels would indicate. This will not be of importance if coverage is very high as in India but may be important in Benin and Malawi. The simulation predictions will be revised in future publications as data accrues during the trial on patterns of compliance to treatment, coverage within each round of MDA and patterns of parasite aggregation (the negative binomial parameter k).

## Conclusions

The current study suggests that heterogeneity will be one of the main factors defining the impact of MDA on disease prevalence in the trial population. Relatively clear indications of successful control at the study arm level are predicted to show a much more varied picture at the cluster level and one that will require further intervention on the cluster scale. Additionally, models fitted to baseline data indicate a wide range of worm aggregation across different clusters, reflecting a heterogeneity in the infectious contact structure at even smaller scales. As prevalence on a large spatial scale is reduced through MDA, heterogeneities at a smaller scale will become more significant, particularly if overall elimination is being attempted. In terms of reaching infected people with chemotherapy, MDA will become less effective, both therapeutically and economically, especially where treatment adherence is poor. Standard methods of surveillance and analysis of data in terms of population-level prevalence will also give a poor picture of the infection state of a community. To achieve elimination, both monitoring and treatment strategies will need take into account parasite aggregation at small scales.

## Supplementary Information

**Additional file 1.** Details of the the sequence of events in the DeWorm3 trial in each country site, up to the current time and as predicted for future events.

**Additional file 2.** Details of the MDA coverage levels achieved in the intervention and control arms of the study in each country site.

**Additional file 3.** Details of the mathematical models used to describe epidemiological, demographic and diagnostic processes and a description of the fitting method.

**Additional file 4.** Details and analysis of the intra-cluster correlation coefficient and its evolution over the course of the trial, as predicted by the simulator.

## Data Availability

The datasets generated and/or analysed during the current study are not publicly available due to blinding requirements of the parent randomized trial but are available from the corresponding author on reasonable request.

## Abbreviations

STH: Soil-transmitted helminths
MDA: Mass drug administration
SAC: School-aged children
MLE: Maximum likelihood estimate
PCR: Polymerase chain reaction
ICC: Intra-cluster correlation coefficient
ITU: independent transmission units
