# Supplementary material for: Forecasting the effectiveness of the DeWorm3 trial in interrupting the transmission of soil-transmitted helminths in three study sites in Benin, India and Malawi
Source: Parasit Vectors. 2021 Jan 20;14:67. doi: 10.1186/s13071-020-04572-7 (PMC7818558; doi:10.1186/s13071-020-04572-7)
Supplement: Supplementary file 1 — Additional file 1. Details of the the sequence of events in the DeWorm3 trial in each country site, up to the current time and as predicted for future events. [file 13071_2020_4572_MOESM1_ESM.pdf]

Additional File 1: Temporal sequence of events

The simulation represents the key features of the trial in each site (rounds of MDA, cross-sectional surveys, sampling, etc) as discrete instantaneous events. Tables 1-6 lists the key events in the life of the trial and their timings within the simulation. For those events that lay in the future at the time of writing, the approximate scheduled dates were used. Within the simulation, when standard of care and MDA occurred in very close succession in a country's intervention arm, we have conflated them into a single MDA event. It is assumed the when MDA within the national program are scheduled in the same month as cross-sectional surveys, the surveys precede the treatment so as not to directly impact and bias the survey (See for example Table 1). National standard of care intervention is suspended in all arms between the last round of MDA and the endline. It is assumed, for the purposes of long-term disease dynamics, that the national standard of care interventions resume after this point and continue indefinitely.

Time	Event	Simulation time point
Dec-Feb, 2018	Baseline survey	2018.10
Feb, 2018	SoC intervention	2018.15
Mar, 2018	MDA: Round 1	2018.2
Aug, 2018	MDA: Round 2	2018.7
Feb, 2019	MDA: Round 3	2019.2
Aug, 2019	MDA: Round 4	2019.7
Feb, 2020	MDA: Round 5	2020.2
Aug, 2020	MDA: Round 6	2020.7
Feb, 2021	Post-intervention XS survey	2021.15
Aug, 2022	Endline XS survey	2022.65
Aug, 2022	SoC intervention	2022.7

Table 1: India site, intervention arm: Past and future events and their time points within the simulation. SoC - Standard of Care; XS - cross-sectional; horizontal lines demarcate years.

Time	Event	Simulation time point
Dec-Feb, 2018	Baseline survey	2018.10
Feb, 2018	SoC intervention	2018.2
Aug, 2018	SoC intervention	2018.7
Feb, 2019	SoC intervention	2019.2
Aug, 2019	SoC intervention	2019.7
Feb, 2020	SoC intervention	2020.2
Aug, 2020	SoC intervention	2020.7
Feb, 2021	Post-intervention XS survey	2021.15
Aug, 2022	Endline XS survey	2022.65

Table 2: India site, control arm: Past and future events and their time points within the simulation. SoC - Standard of Care; XS - cross-sectional; horizontal lines demarcate years.

Time	Event	Simulation time point
Mar-Jun, 2018	Baseline survey	2018.37
Jul-Aug, 2018	MDA: Round 1	2018.6
Nov-Dec, 2018	MDA: Round 2	2018.9
Jun, 2019	MDA: Round 3	2019.45
Nov, 2019	MDA: Round 4	2019.9
Jul-Aug, 2020	MDA: Round 5	2020.45
Nov-Dec, 2020	MDA: Round 6	2020.9
Jun, 2021	Post-intervention XS survey	2021.45
Nov-Dec, 2022	Endline XS survey	2022.9

Table 3: Malawi site, intervention arm: Past and future events and their time points within the simulation. SoC - Standard of Care; XS - cross-sectional; horizontal lines demarcate years.

Time	Event	Simulation time point
Mar-Jun, 2018	Baseline survey	2018.37
Nov, 2018	SoC intervention	2018.85
Nov, 2019	SoC intervention	2019.85
Nov, 2020	SoC intervention	2020.85
Jun, 2021	Post-Intervention Survey	2021.45
Nov-Dec, 2022	Endline Survey	2022.9

Table 4: Malawi site, control arm: Past and future events and their time points within the simulation. SoC - Standard of Care; XS - cross-sectional; horizontal lines demarcate years.

Time	Event	Simulation time point
Apr-May, 2018	Baseline survey	2018.35
Jun, 2018	MDA: round 1	2018.45
Nov, 2018	MDA: round 2	2018.9
Jun, 2019	MDA: round 3	2019.45
Nov, 2019	MDA: round 4	2019.9
Jun, 2020	MDA: round 5	2020.45
Nov, 2020	MDA: round 6	2020.9
Apr, 2021	Post-Intervention Survey	2021.3
Oct, 2022	Endline Survey	2022.8

Table 5: Benin site, Intervention arm: Past and future events and their time points within the simulation. SoC - Standard of Care; XS - cross-sectional; horizontal lines demarcate years.

Time	Event	Simulation time point
Apr-May, 2018	Baseline survey	2018.35
Nov, 2018	SoC Intervention	2018.9
Nov, 2019	SoC Intervention	2019.9
Nov, 2020	SoC Intervention	2020.9
Apr, 2021	Post-Intervention Survey	2021.3
Oct, 2022	Endline Survey	2022.8

Table 6: Benin site, Control arm: Past and future events and their time points within the simulation. SoC - Standard of Care; horizontal lines demarcate years.
