# Supplementary material for: Forecasting the effectiveness of the DeWorm3 trial in interrupting the transmission of soil-transmitted helminths in three study sites in Benin, India and Malawi
Source: Parasit Vectors. 2021 Jan 20;14:67. doi: 10.1186/s13071-020-04572-7 (PMC7818558; doi:10.1186/s13071-020-04572-7)
Supplement: Supplementary file 2 — Additional file 2. Details of the MDA coverage levels achieved in the intervention and control arms of the study in each country site. [file 13071_2020_4572_MOESM2_ESM.pdf]

Additional File 2: Estimation of MDA coverage levels

The effect of both the control and intervention arms of the study on STH prevalence and intensity depend on the level of coverage achieved under MDA and also under the standard of care intervention in each of the sites. Within the simulation, we employ a simple model of treatment compliance, whereby an individual has a constant probability of accepting treatment (stratified by age). Treatment is a random event and uncorrelated with past individual behaviour of with the behaviour of others in the population. The treatment probabilities are calculated from the fraction of individuals receiving treatment in the first round of MDA, using data collected in coverage surveys conducted shortly after the intervention [1]. The datasets typically comprise records from several 10s of thousands of individuals per site. As shown in Table 1, MDA coverage is approximately in the 80-90% range for all sites with little difference between the SAC and adult coverage.

Study site	Coverage	
	0-15 yrs	15+ yrs
India	0.89	0.89
Malawi	0.78	0.78
Benin	0.85	0.81

Table 1: Individual probability of treatment by study site and age category within intervention arms.

Standard of care varies across the country sites in terms of both the age ranges covered and the frequency of implementation. All are broadly focused on school-age children. Levels of coverage within the standard of care arm were not directly available for all country sites. However, they can be indirectly estimated from data collected from the intervention arm. Individuals receiving MDA were asked if they have recently been treated in school or the community. The fraction that respond positively to this question are used as an estimate for the coverage under the national standard of care. In reality, the source of coverage data will include small contributions from individuals who have received treatment through routine antenatal care and other child health services. These have been left in to better reflect actual treatment in the field. It should also be noted that the coverage survey may underestimate the coverage in the control arm if the standard of care intervention overlaps with the survey. Table 2 shows the frequencies, age ranges and coverage estimated in each site.

Study site	Age categories	Coverage	Frequency
India	1-19, 19+	0.6, 0	Twice-yearly
Malawi	5-14, 15-19	0.31, 0.06	Annual
Benin	1-5, 5-14, 15+	0.12, 0.46, 0.04	Annual

Table 2: Frequency, age range and estimated coverage for the standard of care in the three country sites.

References

[1] Ásbjörnsdóttir KH, Ajjampur SSR, Anderson RM, Bailey R, Gardiner I, Halliday KE, et al. Assessing the feasibility of interrupting the transmission of soil-transmitted helminths through mass drug administration: The DeWorm3 cluster randomized trial protocol. PLOS Neglected Tropical Diseases. 2018 jan;12(1):e0006166.
