# Supplementary material for: Forecasting the effectiveness of the DeWorm3 trial in interrupting the transmission of soil-transmitted helminths in three study sites in Benin, India and Malawi
Source: Parasit Vectors. 2021 Jan 20;14:67. doi: 10.1186/s13071-020-04572-7 (PMC7818558; doi:10.1186/s13071-020-04572-7)
Supplement: Supplementary file 3 — Additional file 3. Details of the mathematical models used to describe epidemiological, demographic and diagnostic processes and a description of the fitting method. [file 13071_2020_4572_MOESM3_ESM.pdf]

# Additional File 3: Parameter fitting and diagnostic models

The Kato-Katz diagnostic method for detecting the presence of STH in a host and assessing the intensity of infection has a long history [1, 2]. A standardised volume of material is taken from a stool sample and examined under a microscope to count the type and number of STH eggs. The mean number of eggs counted is taken to be a function of the number of female worms in the host, $n_f$, modified by a density factor mechanism that reduces fecundity through overcrowding,

$$\bar{E} = \lambda n_f \exp(-\gamma n_f) \tag{1}$$

where $\lambda$ is the net egg count per female and $\gamma$ parmeterizes the density-dependent fecundity of egg production. The higher the density of female worms in a host the fewer countable eggs each produces. For hookworm, female worms only produce eggs when fertilized, which means that at least one male worm needs to be present in the host for eggs to be seen. The egg count is highly variable across samples from successive days, having a negative binomial distribution overall [3, 4]. Hence, the probability of counting $E$ eggs from $n_f$ fertilized females in a host is

$$P(E; n_f, \lambda, k_e, \gamma) = \text{NegBin}(E; \bar{E}, k_e). \tag{2}$$

As a result, a zero egg count can arise even when fertilized females are present. Hence, for each individual in a sample, the quantity $1 - P(0; n_f, \lambda, k_e, \gamma)$ gives the probability of getting a positive diagnosis. When applied to all individuals within a sample, this will give a number of positive individuals and hence a prevalence estimate. Values for the paramters $\lambda$ and $k_e$ were investigated in earlier fitting work on hookworm prevalence and intensity [5]. Equations 2 and 1 give a probabilistic relationship between baseline egg count data and modelled quantities such as the distribution of female worms in the host population and serves as the basis for the construction of a likelihood function for the data.

Although the simulator is a stochastic model, we use a simplified deterministic version with two age classes and the same parameters to construct a likelihood for the data.

$$\frac{dM_1}{dt} = L\beta_1 - \sigma M_1, \tag{3}$$

$$\frac{dM_2}{dt} = L\beta_2 - \sigma M_2, \tag{4}$$

$$\frac{dL}{dt} = \psi\left(\pi_1 M_1 f(M_1; \theta) + \pi_2 M_2 f(M_2; \theta)\right) - \mu L. \tag{5}$$

Here, the subscripts 1 and 2 represent school-age children and adult classes, respectively. The variable $M$ is the mean worm burden in the age class and $L$ the quantity of infectious material in the environmental reservoir. Worms are assumed to have a negative binomial distribution across hosts with an aggregation parameter, $k$. Parameters $\pi_i$ are the fraction of the population age in group $i$; $\Sigma\pi_i = 1$. The parameter $\psi$ controls the intensity of the transmission cycle and is proportional to the reproductive ratio, $R_0$. The function $f(M)$ is as defined in equations 6 and 7;

$$f(M) = \left(1 + \frac{M(1-z)}{k}\right)^{-(k+1)} \phi(M) \tag{6}$$

and $\phi(M)$ is the mating probability factor,

$$\phi(M) = 1 - \left(\frac{1 + M(1-z)/k}{1 + M(2-z)/k}\right)^{(k+1)}. \tag{7}$$

where the parameter $z = \exp(-\gamma)$. The flux term between age classes is neglected, given the short lifespan of the worm compared to the width of the age classes. This also allows for closed form solutions for the equilibrium state of the model which make fitting more efficient. We assume that the parasite burden is in equilibrium at the baseline. Although this may not be the case, due to previous rounds of MDA, levels of prior coverage are highly uncertain and likely to be quite low. Previous fitting work which included treatment rounds prior to baseline in the Tumikia study found minimal impact from it [5].

At equilibrium, the solution of the model 3 is given by

$$\Lambda_i = L^* \beta_i, \quad M_i^* = \frac{\Lambda_i}{\sigma} = \frac{L^* \beta_i}{\sigma}$$

The age-dependent contact rates, $\beta_i$, are normalised such that $\Sigma_i \pi_i \beta_i = 1$. This causes the definition of $L$ to be the mean FOI experienced by an individual in the population. As a result,

$$\beta_i = \frac{\Lambda_i}{\pi_1 \Lambda_1 + \pi_2 \Lambda_2}, \tag{8}$$

$$L^* = \pi_1 \Lambda_1 + \pi_2 \Lambda_2, \tag{9}$$

$$R_0 = \frac{\psi z}{\sigma \mu} \tag{10}$$

The parameter $\psi$ (which also defines $R_0$) is defined by

$$\psi \left(\pi_1 M_1 f(M_1; \theta) + \pi_2 M_2 f(M_2; \theta)\right) = \mu L*$$

The probability of a given egg count data from an individual from a population with mean worm burden, $M^*$, is given by summing over all possible worm burdens of an individual,

$$P(E_i; M^*, \lambda, k_e, \gamma) = \sum_{n_f=0}^{\infty} P(E; n_f, \lambda, k_e, \gamma) NB(n_f; M^*, k) \tag{11}$$

In reality, we can approximate this distribution with a single negative binomial for the egg count in terms of $M^*$ and the other parameters by matching its mean and variance with that of equation 11, which can be calculated in closed form (see Appendix of [5] for details). As a result, we write the likelihood for the individual egg counts, $E_i$ as

$$\mathcal{L}(\{E\}; M^*, \lambda, k_e, \gamma) = \prod_i P(E_i; M_i^*, \lambda, k_e, \gamma) \tag{12}$$

where $M_i^*$ is the equilibrium mean burden for the $i_{th}$ individual.

The likelihood is subject to two priors. The first arises from considerations of the stability of the model 3. It is well known that sexual reproduction of the parasite within the

host introduces a 'breakpoint' into the dynamics of parasite transmission, such that when parasites are too scarce for enough mating pairs in the host population, the parasite population collapses [6]. In terms of the model, this situation arises when the determinant of the Jacobian of the endemic solution, $|J|$, is positive. The determinant is given by

$$|J| = -\sigma \left( \sigma\mu - \psi \left( \beta_1 \frac{\partial Q}{\partial M_1} + \beta_2 \frac{\partial Q}{\partial M_2} \right) \right) \tag{13}$$

where $Q = \pi_1 M_1 f(M_1;\theta) + \pi_2 M_2 f(M_2;\theta)$ and $\theta$ represents the other parameters. Hence the first prior, $\pi_J$, is given by

$$\pi_J(M_1, M_2, \theta) = \begin{cases} 1, & |J| < 0 \\ 0, & \text{otherwise} \end{cases}$$

A further prior arises from the need to avoid very large worm burdens. Given the highly skewed nature of the naturally-occurring distributions of worms within hosts, it is hard to define an upper limit. However, work by Anderson and Schad in populations with high prevalence of hookworm indicate a maximum of around 150 worms within a large number of expulsions [3]. To incorporate this information, we define a critical value, $w_f^+$, of female worms, such that the 95% quantile of the negative binomial distribution of worms in an age group should be below $w_f^+$. We let $w_f^+ = 60$. Given the largely independent nature of the two age classes, we write the maximum worm prior, $\pi_w(M_1, M_2, k) = \pi_w(M_1, k; w_f^+, \alpha, \Delta)\pi_w(M_2, k; w_f^+, \alpha, \Delta)$ where

$$\pi_w(M_i, k; w_f^+, \alpha, \Delta) = \frac{1}{1 + f(M_i, k; w_f^+, \alpha, \Delta)}$$

and

$$f(M_i, k; w_f^+, \alpha, \Delta) = \exp\{-(q_{NB}(\alpha; M_i/\sigma, k) - w_f^+)/\Delta\}$$

where $q_{NB}$ is the quantile function of the negative binomial. That is, the sharp transition generated by the critical worm burden $w_f^+$ is 'smoothed' by a logistic function. The quantities $w_f^+ = 60, \alpha = 0.95, \Delta = 2$ are hyper-parameters.

For each cluster in each study arm of each country site, we fit force of infection for each age class, $\Lambda_i$, and the aggregation of the worms in the host, $k$. We take these parameters to represent the heterogeneity in demographic and epidemiological processes that characterise each cluster. From these, values for $R_0$, mean worm burden and other quantities can be calculated as described. Fig 1 shows the MLE values and 90% credible interval of the aggregation parameter, $k$, and the estimated reproduction number $R_0$ for each site, as fitted to the baseline data. Within the simulator, study arms in each country are constructed from the relevant clusters with parameters sampled from the appropriate distributions. Each cluster in each country site is fitted independently to data from the baseline cross-section. The general pattern of fitted aggregation dropping linearly with decreasing prevalence and $R_0$ increasing with deceasing prevalence is seen in the clusters in both India and Malawi. However, the low prevalence and high degree of aggregation encountered in the Benin baseline data make the data difficult to fit to. It is clear that the parameter $R_0$ has a highly skewed distribution, with MLE value very low and falling outside the 90% credible interval, which reaches very

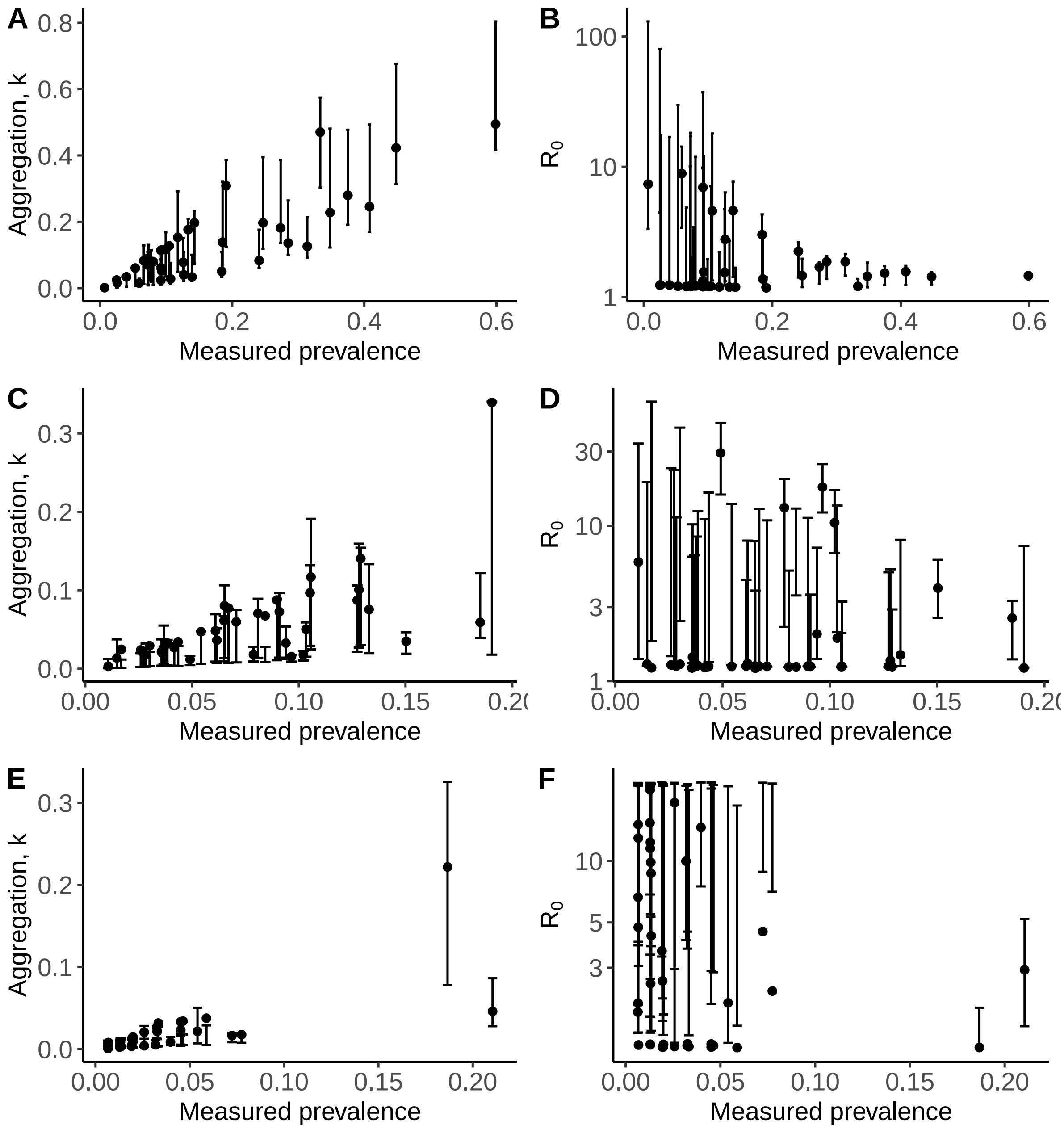


Figure 1: Distributions of fitted parameter values by country site and individual cluster (A and B - India, C and D - Malawi, E and F - Benin). Panels A, C, E show the distributions of worm aggregation, $k$ against measured cluster prevalence and panels B, D, F show the distributions of the basic reproduction number, $R_0$. Dots mark the MLE value and bars represent the 90% credible interval for the parameter.

high values. In this case, an upper limit prior on $R_0$ has been added to prevent very high values.

Other parameter values are shared among all clusters and drawn from the literature. Parameters are listed in Table 1. Worm density dependence describes the effect of the population of worms in an individual on the egg output of individual fertilised female worms, as measured by a standard Kato-Katz slide. The higher the worm burden, the lower the per capita output of eggs. Output of eggs (as counted by Kato-Katz) is highly variable across repeated samples on successive days. The distribution of counts is approximately negative binomial with aggregation parameter, $k_e$. The reservoir timescale is the mean effective survival period for infectious material in the environment.

| Parameter | Symbol | Value | Source |
|---|---|---|---|
| Worm density dependence | $\gamma$ | 0.01 | [5] |
| Mean egg output/female | $\lambda$ | 2.5 eggs/female | [5] |
| Egg aggregation | $k_e$ | 0.8 | [5] |
| Mean worm lifespan | $1/\sigma$ | 2 years | [7] |
| Reservoir timescale | $1/\mu$ | 2 weeks | [8] |
| Drug efficacy | $e_f$ | 0.94 | [9] |

Table 1: Global fixed parameter values used by all clusters in all sites with sources.

# References

[1] Martin LK, Beaver PC. Evaluation of Kato thick-smear technique for quantitative diagnosis of helminth infections. The American journal of tropical medicine and hygiene. 1968 may;17(3):382–91.

[2] Dunn FL. The TIF direct smear as an epidemiological tool; with special reference to counting helminth eggs. Bulletin of the World Health Organization. 1968 jan;39(3):439–49.

[3] Anderson RM, Schad GA. Hookworm burdens and faecal egg counts: an analysis of the biological basis of variation. Transactions of the Royal Society of Tropical Medicine and Hygiene. 1985;79(6):812–825.

[4] de Vlas SJ, Nagelkerke NJ, Habbema JD, van Oortmarssen GJ. Statistical models for estimating prevalence and incidence of parasitic diseases. Statistical methods in medical research. 1993 jan;2(1):3–21.

[5] Truscott JE, Ower AK, Werkman M, Halliday K, Oswald WE, Gichuki PM, et al. Heterogeneity in transmission parameters of hookworm infection within the baseline data from the TUMIKIA study in Kenya. Parasites & Vectors. 2019 dec;12(1):442.

[6] Anderson RM, May RM. Helminth infections of humans: mathematical models, population dynamics, and control. Advances in parasitology. 1985;24:1–101.

[7] Hoagland KE, Schad GA. Necator americanus and Ancylostoma duodenale: life history parameters and epidemiological implications of two sympatric hookworms of humans. Experimental parasitology. 1978 feb;44(1):36–49.

[8] Truscott JE, Turner HC, Farrell SH, Anderson RM. Soil-Transmitted Helminths. In: Advances in Parasitology. vol. 94. Elsevier; 2016. p. 133–198.

[9] Vercruysse J, Behnke JM, Albonico M, Ame SM, Angebault C, Bethony JM, et al. Assessment of the Anthelmintic Efficacy of Albendazole in School Children in Seven Countries Where Soil-Transmitted Helminths Are Endemic. PLoS Neglected Tropical Diseases. 2011 mar;5(3):e948.
