# Supplementary material for: Forecasting the effectiveness of the DeWorm3 trial in interrupting the transmission of soil-transmitted helminths in three study sites in Benin, India and Malawi
Source: Parasit Vectors. 2021 Jan 20;14:67. doi: 10.1186/s13071-020-04572-7 (PMC7818558; doi:10.1186/s13071-020-04572-7)
Supplement: Supplementary file 4 — Additional file 4. Details and analysis of the intra-cluster correlation coefficient and its evolution over the course of the trial, as predicted by the simulator. [file 13071_2020_4572_MOESM4_ESM.pdf]

Additional File 4: Intra-cluster correlation coefficient before and after the trial

Standard sample size calculations for cluster-based trials use the intra-cluster correlation coefficient (ICC) to summarise the heterogeneity among clusters. Combined with the mean prevalence across an study arm, the expected variance in prevalence in the arm can be calculated [1, 2]. In order to use the formalism, it is necessary to estimate the value of ICC at the endpoint, either from pre-trial data or from elsewhere. The simulator allows us to calculate the distribution of the intra-cluster correlation coefficient at the baseline and endline of the trial directly.

Figure 1A shows the distribution of ICC at the baseline and endline of the trial. Mean ICC and also the variance in the estimate drop across the rounds of MDA from an initial mean of 0.15 to around 0.04. A very simple theory for the change in ICC is to assume that the trial results in a proportional drop in prevalence for each cluster. If the reduction in prevalence is by a factor ϵ, then the variance in the mean prevalence will drop by ϵ^2. Given a mean prevalence across clusters of μ_0 and ICC of ρ_0 at baseline, the predicted values at endline are

$$\mu = \epsilon\mu, \quad \rho = \rho_0 \frac{\epsilon(1-\mu_0)}{1-\epsilon\mu_0}.$$

For low values of initial prevalence, ICC drops in an almost linear fashion by a factor ϵ. The relationship is shown in Figure 1B. The value of ICC predicted at the endpoint by this simple approach matches the simulated result well.

References

[1] Hayes RJ, Bennett S. Simple sample size calculation for cluster-randomized trials. International journal of epidemiology. 1999 apr;28(2):319–26.

[2] Hayes RJ, Moulton LH. Cluster randomised trials. Chapman and Hall/CRC; 2017.

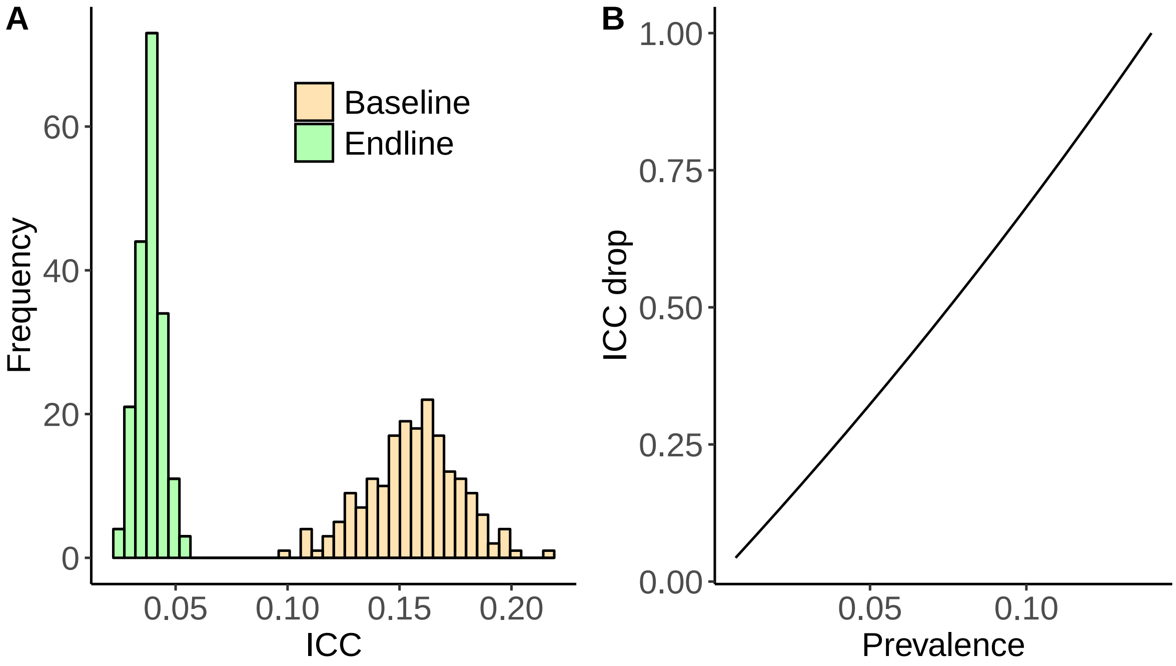

Figure 1: A) Simulated distribution of ICC values calculated at baseline and endline of the intervention arm at the India site. B) The predicted relationship between endline prevalence and the fractional change in ICC across the trial according to the simple model (assuming $\mu_0 = 0.14$). For consistency, Kato-Katz diagnostic is used at baseline and endline for prevalence.
